# Perfluorocarbon Nanodroplets for Dual Delivery with Ultrasound/GSH-Responsive Release of Model Drug and Passive Release of Nitric Oxide

**DOI:** 10.3390/polym14112240

**Published:** 2022-05-31

**Authors:** Moonhyun Choi, Arman Moini Jazani, Jung Kwon Oh, Seung Man Noh

**Affiliations:** 1 Research Center for Green Fine Chemicals, Korea Research Institute of Chemical Technology, Ulsan 44412, Korea; mhchoi.yonsei@gmail.com; 2 Department of Chemistry and Biochemistry, Concordia University, Montreal, QC H4B 1R6, Canada; ajazani@andrew.cmu.edu

**Keywords:** perfluorocarbon, nanodroplets, NO delivery, drug delivery, glutathione, ultrasound, stimuli-responsive degradation, enhanced drug release

## Abstract

Nitric oxide (NO) plays a critical role as an important signaling molecule for a variety of biological functions, particularly inhibiting cell proliferation or killing target pathogens. To deliver active radical NO gaseous molecule whose half-life is a few seconds in a stable state, the design and development of effective exogenous NO supply nanocarriers are essential. Additionally, the delivery of desired drugs with NO can produce synergistic effects. Herein, we report a new approach that allows for the fabrication of dual ultrasound (US)/glutathione (GSH)-responsive perfluorocarbon (PFC) nanodroplets for the controlled release of model drug and passive release of safely incorporated NO. The approach centers on the synthesis of a disulfide-labeled amphiphilic block copolymer and its use as a GSH-degradable macromolecular emulsifier for oil-in-water emulsification process of PFC. The fabricated PFC nanodroplets are colloidally stable and enable the encapsulation of both NO and model drugs. Encapsulated drug molecules are synergistically released when ultrasound and GSH are presented, while NO molecules are passively but rapidly released. Our preliminary results demonstrate that the approach is versatile and can be extended to not only GSH-responsive but also other stimuli-responsive block copolymers, thereby allowing for the fabrication of broad choices of stimuli-responsive (smart) PFC-nanodroplets in aqueous solution for dual delivery of drug and NO therapeutics.

## 1. Introduction

Nitric oxide (NO) is an important signaling molecule for a variety of biological functions and also participates in various biomedical processes such as cardiac contractility [1,2], vasodilation [3], antibacterial effects [4], cell proliferation [5,6] and anti-cancer effects [7,8]. However, NO is a diatomic free radical (uncharged molecule) containing an unpaired electron. NO remains stable as long as it is not exposed to reactive species; however, it reacts very rapidly with oxygen and water to form nitrous acid (HNO2) or nitrogen dioxide (NO2) [9,10,11]. These nitrogen oxide species (NOx) are known to be involved in protein oxidation reactions under physiological conditions [12]. Hence, it is required to minimize the contact and reaction of NO with oxygen for the successful delivery of NO gas.

Perfluorocarbon (PFC) liquids, which are immiscible with aqueous solutions, can dissolve large quantities of gases. PFCs are very stable because of the high strength of the carbon-fluorine bond, one of the strongest bonds in organic chemistry [13]. The electronegativity of fluorine atom imparts partial ion properties to carbon and fluorine atoms through partial charges, which shortens and strengthens C-F bonds through covalent interactions [14]. The high solubility of gas in PFC is attributed to the weak intermolecular interaction of these carbon fluoride fluids. Taking these advantages, PFCs have been used as potential carriers of gaseous molecules, including NO. 

A promising approach toward the delivery of NO involves the fabrication of nanometer-sized droplets (nanodroplets) containing PFC liquid cores where NO molecules could be encapsulated. The approach has explored an oil-in-water emulsification method with an aid of extra surface-active agents to form colloidally-stable PFC nanodroplets dispersed in aqueous solution. Mostly small molecular weight surfactants [15,16], phospholipids [17,18] and recently a block copolymer consisting of commercially-available Pluronics [19] are surface-active agents that have been used. The formed NO-containing PFC nanodroplets could encapsulate hydrophobic drug molecules for dual delivery. However, most of them could lack the ability to degrade and thus the controlled release of encapsulated drug molecules responded to biological environments.

**Scheme 1:**
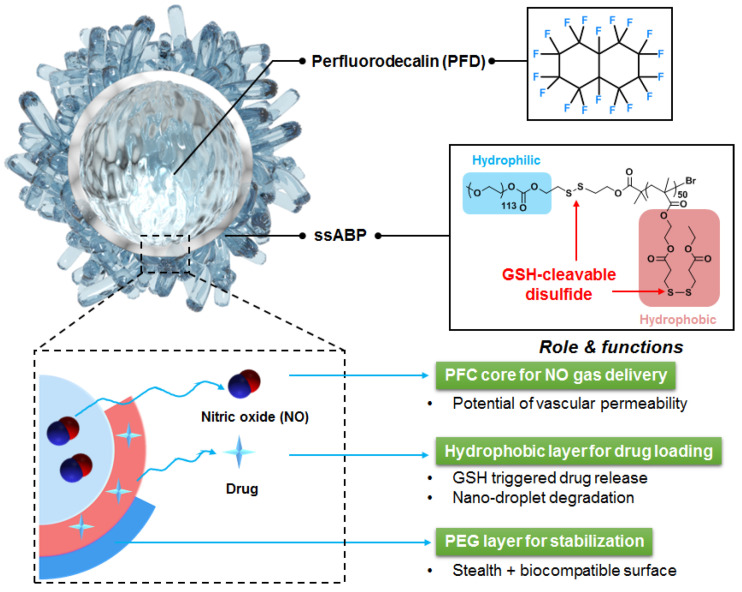
Illustration of the new concept that centers on the use of an SRD-exhibiting ABP (here ssABP) as a macromolecular stabilizer to fabricate colloidally-stable, smart nanodroplets of a PFC liquid core stabilized with ssABP for controlled/enhanced release of NO and model drugs.

The stimuli-responsive degradation (SRD) platform has been tremendously exploited in the construction of smart amphiphilic block copolymers (ABPs) and their self-assembled micelles (nanoassemblies) for controlled/enhanced drug delivery [20,21,22,23,24,25,26]. SRD-exhibiting ABPs are designed with labile (or cleavable) linkages, particularly disulfide linages to achieve reductive degradation [27,28,29,30,31,32]. In response to glutathione (a tripeptide as a cellular reducing agent) found in cells [33,34,35], disulfide-labeled ABPs and their nanoassemblies degrade (or disintegrate) upon the cleavage of disulfide bonds, leading to enhanced drug release in aqueous and biological environments. Given the features of the SRD platform, we have explored a new approach that centers on the integration of the SRD platform in the fabrication of stimuli-responsive degradable (or smart) nanodroplets of PFD liquid cores in aqueous solution. The approach involves the synthesis and use of SRD-exhibiting ABPs as smart macromolecular emulsifiers to stabilize PFC-nanodroplets through the emulsification method. As illustrated in Scheme 1, SRD-ABP-stabilized PFC-nanodroplets enable the encapsulation of both NO and drug molecules and the controlled/enhanced release of encapsulated drug molecules when external triggers are applied. 

In this study, we demonstrate the feasibility of the new approach with the synthesis of a GSH-degradable ABP (ssABP) to fabricate dual ultrasound/GSH-responsive PFC nanodroplets for dual delivery of a model drug and NO therapeutics. The ssABP consists of hydrophilic poly(ethylene glycol) block connected through a junction disulfide linkage with a polymethacrylate block having disulfide pendants. These disulfide linkages could be cleaved in the presence of cellular GSH. Given the synthesis of ssABP by a controlled radical polymerization, we examined ssABP as a smart macro-emulsifier for the emulsification process with both formulation and processing parameters that significantly influenced the fabrication and colloidal stability of PFC-nanodroplets stabilized with ssABP. Furthermore, we studied the encapsulation and passive release of NO molecules as well as the synergic release of an encapsulated model drug in response to dual GSH and ultrasound stimuli. 

## 2. Materials and Methods

### 2.1. Materials and Instrumentation

Perfluorodecalin (PFD, 95%), phosphate buffered saline (PBS) solution (pH = 7.4), Nile Red (NR), Glutathione (GSH) and *N,N-*dimethylformamide (DMF) were purchased from Sigma-Aldrich (St. Louis, MO, USA). ssABP was synthesized and purified as described in our previous publication [36].

1H-NMR spectroscopy was used for structure determination and conversion analysis using a 500 MHz Varian spectrometer (Santa Clara, CA, USA).

United States. Gel permeation chromatography (GPC) with poly(methyl methacrylate) standards was conducted for molecular weight analysis of ssABP with a Viscotek VE1122 pump and a refractive index (RI) detector were conducted. Dynamic light scattering (DLS) analysis was performed for size and size distribution analysis of the formed nanodroplets with a Malvern Instruments Nano ZS90 equipped with a 633 nm He-Ne gas laser at a fixed scattering angle of 175° at 25 °C. 

### 2.2. General Procedure for Emulsification Process to Prepare Qqueous PFD Nanodroplet Dispersion

Aliquots of the purified, dried ssABP (3–36 mg) dissolved in DMF (0.8 mL) were mixed with PBS (5 mL) to form mixtures with the concentration of ssABP to be 0.5–3 mg/mL. After the addition of different volumes of PFD (40, 80, and 160 µL), the resulting mixtures were subjected to vigorous sonication using a probe-type ultrasonic wave homogenizer (model 185, Branson Ultrasonics, Danbury, Connecticut,, USA) under the conditions (5 s ON and 5 s OFF; 10 cycle) in an ice bath to produce nanosized emulsion droplets. They were then subjected to intensive dialysis using a dialysis tubing with MWCO = 100,000 g/mol against deionized water (500 mL) twice for 24 h, yielding aqueous dispersions of PFD-nanodroplets stabilized with ssABP. 

### 2.3. Preparation of NR-Loaded PFD-Nanodroplets

A similar procedure to fabricate bare PFD-nanodroplets was used except for the use of NR (90 mg) mixed with ssABP (18 mg). NR was used as a hydrophobic model drug for studying a release behavior of molecules.

### 2.4. Release of NR in Response to GSH and Ultrasound

For GSH treatment, an aliquot of aqueous 100 mM GSH solution (0.3 mL) was mixed with an aqueous dispersion of NR-loaded PFD-nanodroplets (2.7 mL). The resulting mixture was incubated at room temperature and its fluorescence spectra were recorded using photoluminescence spectroscopy (FP-8300, Jasco, Japan) at given times. For ultrasound treatment, an ultrasound wave was generated by an ultrasound machine (HS-502, Hanil-TM). It applies constant supersonic waves for 2 min with 1 MHz of frequency and 3 w/cm^2^ of strength. The fluorescence spectra of NR was recorded at given times. 

### 2.5. NO Loading and Release

Aliquots of PFD-nanodroplets were degassed in a high-pressure reactor by bubbling pure argon for 2 min three times at room temperature. They were then equilibrated with NO gas for 2 h (1 bar, 25 °C). Real-time NO release measurements were performed using a chemiluminescence NO analyzer (NOA, Sievers 280i, GE Analytical Instruments, Boulder, CO, USA).

## 3. Results and Discussion

**Figure 1:**
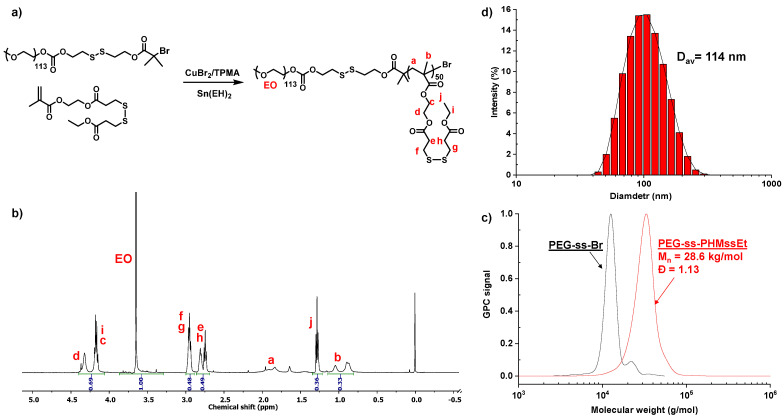
Synthetic scheme (**a**), ^1^H-NMR spectrum in CDCl_3_ (**b**), GPC chromatogram (**c**), and self-assembled micelles (**d**) of a well-defined ssABP.

Figure 1a depicts our approach to synthesizing a well-defined ssABP with narrow molecular weight distribution. The approach utilizes atom transfer radical polymerization (ATRP), a well-established controlled radical polymerization technique [37,38], for a methacrylate monomer having a pendant disulfide linkage (called HMssEt), initiated with a poly(ethylene glycol)-based disulfide-functionalized bromine macroinitiator (PEO-ss-Br). The synthesized ssABP consists of a hydrophobic PHMssEt block having pendant disulfide linkages, which is connected through a disulfide linkage with a hydrophilic PEG block, thus a PEG-ss-PHMssEt diblock copolymer. It is characteristic due to the position of GSH-responsive cleavable disulfide linkages both at the junction of PEG and PHMssEt blocks and as pendants in the hydrophobic PHMssEt block. Its ^1^H-NMR spectrum in Figure 1b shows the presence of the PEG block at 3.5–3.7 ppm and the PHMssEt block at 0.8–1.2 ppm. Their integral ratio with the degree of polymerization of PEG = 113 (corresponding to its molecular weight of 5 kg/mol) allows for the determination of DP of the PHMssEt block to be 50. Our gel permeation chromatography (GPC) analysis confirms that ssABP had the number average molecular weight (M_n_) = 28.6 kg/mol with dispersity (Đ) = 1.13 (Figure 1c). 

The ssABP is amphiphilic and its critical micelle concentration (CMC) was determined to be 17 µg/mL in water by a fluorescence spectroscopy technique with an NR probe. This technique relies on the fact that fluorescence becomes intense when NR molecules are present (or encapsulated) in hydrophobic cores, while it is lower in an aqueous solution. The CMC can be determined when the fluorescence intensity of NR begins to abruptly increase. At concentrations above its CMC, ssABP self-assembles in aqueous solution to form colloidally-stable nanoassemblies with nanometer-sized diameters. Figure 1d shows the DLS diagram of the nanoassemblies with the diameter to be 114 nm at 1.4 mg/mL. The detailed synthesis, characterization, and aqueous micellization of ssABP is reported in our previous publication [36].

**Figure 2:**
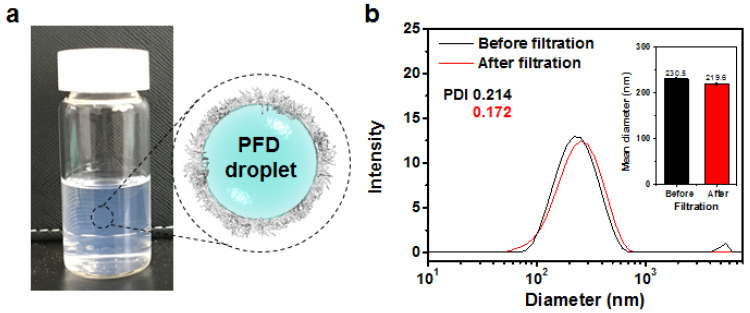
Digital image and schematic representation of aqueous dispersion of PFD nanodroplets stabilized with ssABP in aqueous PBS solution (**a**) and DLS diagrams and mean diameters (inset) of nanodroplets prepared at 18/0.31 wt/wt of ssABP/PFD in PBS solution before and after filtration (**b**).

Given the amphiphilicity toward aqueous self-assembly, we explored the ability of ssABP as a macromolecular emulsifier (or stabilizer) in our emulsification method to fabricate nanometer-sized droplets (nanodroplets) of fluorocarbon stabilized with ssABP in PBS at pH = 7.4. PFD is a fluorocarbon with its molecular formula of C_10_F_18_. It is chemically and biologically inert as well as FDA-approved for use as an ^19^F-MRI contrast enhancement agent and blood substitute. With our choice of PFD, the first step in our emulsification process involves the formation of the mixtures of an organic solution of ssABP dissolved in DMF with aqueous PBS solution. The resulting mixtures appeared to be blue-tinted, suggesting the formation of nanoassemblies in a mixture of DMF/water (1/5 wt/wt). The second step involves the addition of PFD. The resulting mixtures were subjected to sonication using a probe-type ultrasonic wave homogenizer, followed by intensive dialysis against water, yielding colloidally-stable PFD-nanodroplets stabilized with ssABPs (Figure 2a). 

Figure 2b shows the DLS diagrams of ssABP-stabilized PFD-nanodroplets prepared with 18/0.31 wt/wt of ssABP/PDF in PBS solution. Before the purification by filtration, the diagram shows a major population (>73% by volume) with an average diameter = 230.3 nm by intensity as well as a small population (<25% by volume) of large aggregates with diameter >1 µm. Such large aggregates appeared to be easily removed by simple filtration with a PES filter (400 nm pore-sized). Furthermore, the size distribution after filtration did not appear to be significantly changed with an average diameter = 219.6 nm.

**Figure 3:**
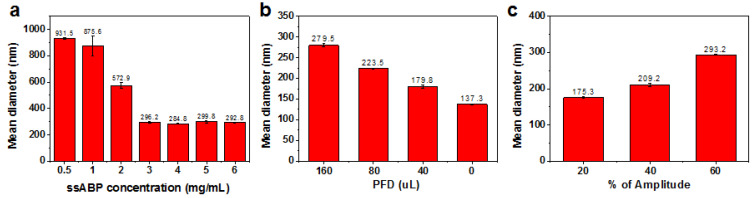
Exploration of formulation and processing parameters that influence the emulsification and fabrication of PFD-nanodroplets. Mean diameter by intensity over ssABP amount (**a**), PFD amount (**b**), and amplitude for ultra-sonication (**c**).

We then investigated both important formulation and processing parameters that influence the emulsification and fabrication of PFD-nanodroplets. An important formulation parameter is the amount of ssABP stabilizer compared to PFD droplets. With the fixed amount of PFD, the amounts of ssABP were varied, as the varying wt ratio of ssABP/PFD from 3/0.31 to 36/0.31. As seen in Figure 3a, the droplets prepared with the wt ratio = 3/0.31 (0.5 mg/mL) had a diameter >900 nm. Such a large diameter could be attributed to the lower amount of ssABP that would not be enough to stabilize hydrophobic PFD droplets. When the amount of ssABP increased, the diameter decreased to 296.2 nm. Upon its further increase, the diameter remained unchanged. This result suggests that ssABP copolymer could be adsorbed on the surfaces of nanodroplets to reduce interfacial tensions, thus promoting steric stabilization. 

Another formulation parameter is the amount of PFD compared to ssABP stabilizer. With the fixed amount of ssABP, the amount of PFD was varied as the wt ratio of PFD/ssABP. As seen in Figure 3b, the diameter of the formed nanodroplets was significantly decreased by 100 nm from 279.5 nm to 179.7 nm, when the amount of PFD decreased from 160 to 40 uL in the recipe. Note that the diameter of the nanodroplets without PFD was 137.5 nm, which is somewhat larger than that (114 nm) of self-assembled micelles. The plausible reason is due to sonication after aqueous micellization in the emulsification process. These results suggest that the droplet sizes rely on the amount of PFD and the nanodroplets prepared with 40 uL of PFD looked to be promising for further experiments.

Further, amplitude for ultra-sonication as one of the processing parameters was investigated. The amplitude during sonication is the distance at which the probe vibrates by a given vibration energy. The larger the set amplitude, the larger the amount of energy delivered to the liquid. With a 13 mm diameter probe, the vibration distance of the probe can be changed depending on percentages of amplitude. Here, the vibration distances of the probe varied with 22.8, 45.6, and 68.4 um corresponding to 20%, 40% and 60% amplitude, respectively. As seen in Figure 3c, the diameter of the formed nanodroplets increased from 175.3 to 293.2 nm with an increasing amplitude from 20% to 60%. 

These results suggest that both the formulation and processing parameters (amount of ssABP and PFC as well as amplitude) significantly influence the fabrication of PFD-nanodroplets stabilized with ssABP. Further, they are promising because they can allow us to tune the sizes of the nanodroplets. For intravenous delivery, it is essential to design nanodroplets with their optimal size to be 30–200 nm because they have enhanced blood circulation time. The nanomaterials with their diameters being larger than 200 nm could be easily removed by mononuclear phagocytic systems mediated by cells in liver, spleen, and bone marrow during blood circulation. Those with their sizes being smaller than 30 nm could be facilely removed through renal clearance (or kidney filtration) or be extravasated to normal tissues. 

**Figure 4:**
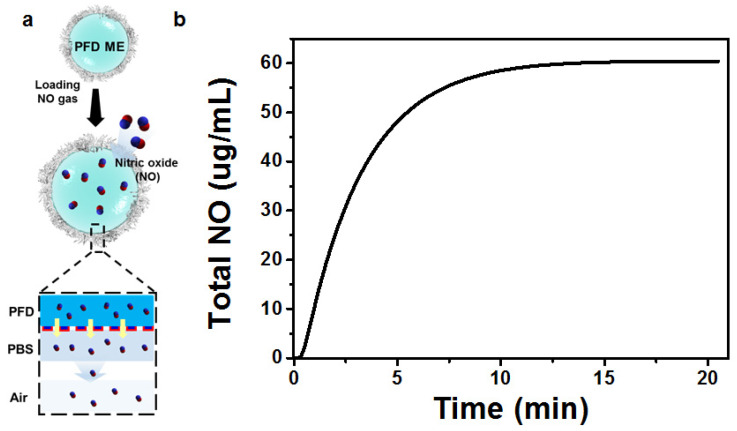
Schematic illustration of encapsulation of NO in PFC liquid cores of PFD nanodroplets stabilized with ssABP and its release (**a**) and accumulated amount of NO released from PFD-nanodroplets over time (**b**).

After our systematic studies on the emulsification process that allows for the fabrication of ssABP-stabilized PFD-nanodroplets, we examined the encapsulation of NO in PFD liquid cores and its release from the nanodroplets. The formed PFD-nanodroplets with the optimal size consist of a hydrophobic PFD liquid core, surrounded by an ssABP shell. The stabilizing shell could be composed of a hydrophobic PHMssEt layer which is absorbed on PFD surfaces and water-soluble PEG coronas. In our ssABP-stabilized PFD nanodroplets, hydrophobic PHMssEt polymer blocks of ssABP are positioned at interfaces between PFD liquid cores and water, which could circumvent any contacts of NO in PFD cores with oxygen in water. As a consequence, NO gas scarcely reacts with O_2_ and could be safely stored in the PFD cores of PFD-nanodroplets, yielding NO-encapsulated PFD-nanodroplets stabilized with ssABP (Figure 4a). These nanodroplets could store NO molecules. It can be anticipated that they can deliver NO molecules to desired organs or tissues after intravenous injection in the blood. As seen in Figure 4b, the encapsulated NO is rapidly released over time. The amount of NO released from PFD-nanodroplets turned out to be 2.0 μmol/mL in 18 min.

Further, we investigated the loading and stimuli-responsive enhanced release of a model drug to see its feasibility for biomedical applications, particularly smart delivery. Hydrophobic guest molecules could be encapsulated in the hydrophobic PHMssEt layer and the PFD core. With our choice of NR as a model drug, the similar protocol of emulsification in the presence of NR was examined to fabricate NR-loaded PFD-nanodroplets stabilized with ssABPs. 

**Figure 5:**
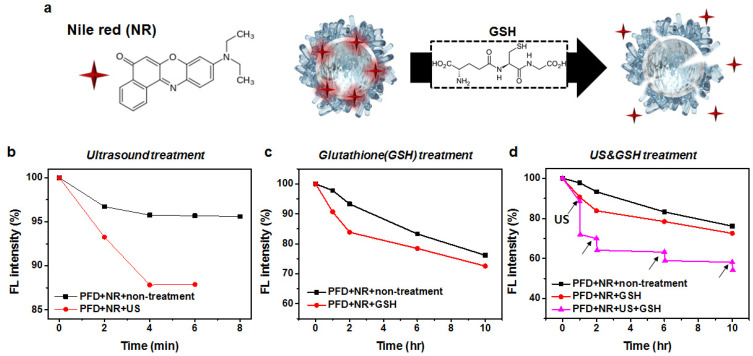
Schematic illustration of GSH-responsive degradation of NR-loaded PFD-nanodroplets stabilized with ssABP for enhanced release of encapsulated NR molecules (**a**) as well as normalized fluorescence intensity of NR over incubation time when ultrasound was applied (**b**), in the presence of 10 mM GSH (**c**), and under a dual ultrasound and 10 mM GSH condition (**d**), compared with no stimuli.

They were then evaluated for the enhanced release of encapsulated NR molecules in response to GSH and ultrasound stimuli (Figure 5a). Their aliquots were either subjected to ultrasound or incubated with 10 mM GSH. The fluorescence intensities of NR were monitored over time. This method utilizes the low solubility of NR in an aqueous solution. When NR-loaded nanodroplets are destabilized to be precipitated, NR molecules are released to aqueous solution, which are eventually precipitated, leading to a decrease in fluorescence intensity. 

Figure 5b shows the results with ultrasound stimulus. With no ultrasound, the fluorescence intensity of NR slightly decreased by <4% over 8 min. When ultrasound was applied, the intensity was dropped by 12% within 4 min, which could be attributed to the destabilization of the nanodroplets upon ultrasound stimulation. Figure 5c shows the results with 10 mM GSH. The fluorescence intensity of NR decreased over incubation time in the absence and presence of 10 mM GSH. Although the difference of NR release rate did not appear to be significant, the NR release was likely to be faster in 10 mM GSH within 10 h. ssABP is designed with GSH-cleavable disulfide linkages at the block junction and as pendant groups in hydrophobic blocks. Upon the cleavage of these disulfide linkages, ssABP loses its amphiphilicity (e.g., surface-active ability), causing the destabilization or disintegration of PFD nanodroplets, thus leading to the enhanced release of encapsulated drug molecules. Compared with these NR release results in response to a single stimulus (ultrasound or GSH), Figure 5d shows the dramatic release of encapsulated NR when ultrasound was applied in 10 mM GSH solution for 2 min. The intensity was dropped by >40% within 10 h. These results confirm the synergistic release of encapsulated NR in a dual ultrasound and GSH condition.

## 4. Conclusions

The emulsification process with the aid of an ssABP as a GSH-degradable macro-emulsifier allowed for the fabrication of colloidally-stable nanodroplets of PFD liquid cores exhibiting dual responses to GSH and ultrasound. Our systematic investigation revealed that the amounts of ssABP and PFD, as well as sonication pulses, are important formulation and processing parameters that can significantly influence the colloidal stability and size of aqueous PFD-nanodroplets stabilized with ssABP. The fabricated PFD-nanodroplets with optimal diameters enabled us to encapsulate a large amount of therapeutic NO gas in PFD liquid cores with no chemical reactions. Promisingly, they exhibited the synergistic release of an encapsulated model drug upon their degradation caused by the cleavage of disulfide linkages positioned at the block junctions and pendant chains in the presence of 10 mM GSH, combined with ultrasound. Encapsulated NO molecules were passively released in a rapid manner. Our preliminary results suggest that the new approach exploring the synthesis and use of SRD-exhibiting ABPs as degradable macromolecular emulsifiers for the oil-in-water emulsification process is versatile in the development of dual smart (ultrasound/stimulus-responsive) PFC-nanodroplets for dual delivery of model drugs and NO therapeutics.

## Data Availability

Not applicable.
